# Simulation of an SEIR infectious disease model on the dynamic contact network of conference attendees

**DOI:** 10.1186/1741-7015-9-87

**Published:** 2011-07-19

**Authors:** Juliette Stehlé, Nicolas Voirin, Alain Barrat, Ciro Cattuto, Vittoria Colizza, Lorenzo Isella, Corinne Régis, Jean-François Pinton, Nagham Khanafer, Wouter Van den Broeck, Philippe Vanhems

**Affiliations:** 1 Centre de Physique Théorique de Marseille, CNRS UMR 6207, Marseille, France; 2 Hospices Civils de Lyon, Hôpital Edouard Herriot, Service d'Hygiène, Epidémiologie et Prévention, Lyon, France; 3 Université de Lyon; université Lyon 1; CNRS UMR 5558, laboratoire de Biométrie et de Biologie Evolutive, Equipe Epidémiologie et Santé Publique, Lyon, France; 4 Data Science Laboratory, Institute for Scientific Interchange (ISI) Foundation, Torino, Italy; 5 INSERM, U707, Paris F-75012, France; 6 UPMC Université Paris 06, Faculté de Médecine Pierre et Marie Curie, UMR S 707, Paris F75012, France; 7 Computational Epidemiology Laboratory, Institute for Scientific Interchange (ISI) Foundation, Torino, Italy; 8 Laboratoire de Physique de l'Ecole Normale Supérieure de Lyon, CNRS UMR 5672, Lyon, France

## Abstract

**Background:**

The spread of infectious diseases crucially depends on the pattern of contacts between individuals. Knowledge of these patterns is thus essential to inform models and computational efforts. However, there are few empirical studies available that provide estimates of the number and duration of contacts between social groups. Moreover, their space and time resolutions are limited, so that data are not explicit at the person-to-person level, and the dynamic nature of the contacts is disregarded. In this study, we aimed to assess the role of data-driven dynamic contact patterns between individuals, and in particular of their temporal aspects, in shaping the spread of a simulated epidemic in the population.

**Methods:**

We considered high-resolution data about face-to-face interactions between the attendees at a conference, obtained from the deployment of an infrastructure based on radiofrequency identification (RFID) devices that assessed mutual face-to-face proximity. The spread of epidemics along these interactions was simulated using an SEIR (Susceptible, Exposed, Infectious, Recovered) model, using both the dynamic network of contacts defined by the collected data, and two aggregated versions of such networks, to assess the role of the data temporal aspects.

**Results:**

We show that, on the timescales considered, an aggregated network taking into account the daily duration of contacts is a good approximation to the full resolution network, whereas a homogeneous representation that retains only the topology of the contact network fails to reproduce the size of the epidemic.

**Conclusions:**

These results have important implications for understanding the level of detail needed to correctly inform computational models for the study and management of real epidemics.

Please see related article BMC Medicine, 2011, 9:88

## Background

The pattern of contacts between individuals is a crucial determinant for the spread of infectious diseases in a population [1]. The topological structure of the contact network of the population, the presence of people with a much larger number of contacts than the mean value [2-5], the clustering and presence of well-identified communities of people [6-10], and the frequency and duration of contacts [11-13] all have important implications for the spread and control of epidemics. Knowledge of contact patterns is crucial for building and informing computational models of infectious disease transmission [14-23]. Although some of the properties of contact patterns can dramatically affect the model predictions [3-5], little is known about their empirical characteristics, and few experiments have been conducted to collect data on how individuals mix and interact.

The starting point of most modeling approaches is the assumption of homogeneous mixing, which assumes that every individual has an equal probability of contacting other individuals in the population [1]. No heterogeneity in the mixing pattern or in the duration or frequency of the contact is considered, and the dynamic nature of the contacts is disregarded. Going beyond this approximation, various approaches have been proposed to estimate mixing properties between classes of people (for example, social or age classes) using indirect [1] and, more recently, direct [11,24-27] methods. Indirect methods are based on estimating the elements of a 'who acquires infection from whom' (WAIFW) matrix using observed seroprevalence data. In direct methods, each element of a contact matrix is estimated independently from the epidemiologic data. Direct methods rely on data collection about at-risk events via diaries [11,12] or time-use data [2,27]. To date, research on human social interaction has been mainly based on self-reported data. Despite a real improvement in the description of potential contacts with respect to a homogeneous mixing approach, self-report methods involve a limited number of people who provide information on a limited number of snapshots in time (usually 1 day). The obtained data may be subject to uncontrolled bias and a lack of representativeness, because they are not based on objective reports, and because the data collection is performed on a random day and is not longitudinal. These limitations become particularly relevant in the case of contact patterns and infectious diseases transmitted by the respiratory or close-contact routes. For these diseases, all types of social encounters, even random contacts of very short duration (for example, on public transport), may be important for transmission, but are rather difficult to report objectively and exhaustively through a diary method.

New technologies are now available that allow the tracking of proximity to and interactions between individuals [28-37], greatly transforming our ability to understand and characterize social behavior [38]. Detection of contact patterns can rely on objective and unsupervised measures of proximity behavior that can be extended to a large number of people, with high temporal and spatial resolution [28,30], thus overcoming the limitations of self-reported data. Departing from the typical static representation of a network of contacts between individuals [39], it is now possible to describe the dynamic nature of the interactions. Analysis of the dynamics of a contact network needs to incorporate two essential features: (i) variations in the duration and frequency of the contacts between individuals, and (ii) the existence of causality constraints in the possible chains of transmission.

Finally, little is known about the level of detail that should be incorporated in the modeling effort to perform in practice realistic simulations of epidemics spreading in a population. Very coarse descriptions of human behavior, such as the homogeneous mixing hypothesis, leave out crucial elements. Conversely, extremely detailed information may yield a lack of transparency in the models, making it difficult to discriminate the effect of any particular modeling assumption or component.

The aim of this study was to assess the role of the temporal aspects, heterogeneities and constraints of dynamic contact patterns in shaping the dynamics of an infectious disease in a population using data collected during a 2-day medical conference. In this study, we capitalized on the recent development of a data-collection infrastructure that allows the tracking of face-to-face proximity of individuals at a high temporal resolution [28,30]. We used the data collected during a scientific conference to provide temporal information on individual contact events. Such data can be mapped onto a dynamic network of contacts, in which all information on interactions between pairs of individuals, time of occurrence and duration are explicit in the network representation. Along with the explicit dynamic network of contacts, we considered two different projections of the data, defining two types of daily networks that aggregate the empirical data in different ways, which reflect different amounts of available knowledge about the contacts between individuals. We then simulated the spread of an infectious disease over these networks, and highlighted the role that different features of contact patterns and their dynamic aspects played during the course of the simulated outbreak. The results have important implications for identification of the level of detail needed for contact data to adequately and realistically inform modeling approaches applied to public health problems.

## Methods

The ethics committee of Lyon University Hospital approved this study, and all participants gave signed, written informed consent. The data were collected anonymously.

### Data collection platform

Contact network measurements are based on the SocioPatterns RFID platform (http://www.sociopatterns.org) [28,30]. With this method, subjects wear a badge equipped with an active radiofrequency identification (RFID) device (tag). RFID devices engage in bidirectional radio communication at multiple power levels, exchanging packets that contain a device-specific identifier. At low power level, packets can only be exchanged between tags within a radius of 1 to 2 meters [28,30]. This threshold is set to allow detection of a close-contact situation, during which a communicable disease infection can be transmitted, either by airborne transmission through coughing or sneezing, or directly by physical contact. Subjects wear the RFID badges on their chest, so that contacts are recorded only when participants face each other, as the body acts as a shield for the proximity-sensing RF signals. In addition to sensing nearby devices, RFID tags send the locally collected contact information to a number of receivers installed in the environment, which relay this information over a local area network to a computer system used for monitoring and data storage. Proximity scans are performed at random times, and each tag dispatches information to the receivers every few seconds. Time is then coarse-grained over 20 second intervals, during which face-to-face proximity can be assessed with a confidence in excess of 99% [28,30]. This time scale is also adequate to follow the dynamics of social interaction.

All communication (from tag to tag, from tags to receivers, and from receivers to the data storage system) is encrypted. Contact data are stored in encrypted form, and all data management is completely anonymous. Other details on the data-collection infrastructure can be found elsewhere [28,30].

### Data collection in this study

Participants attending the 2009 Annual French Conference on Nosocomial Infections (http://www.sf2h.net/) were asked to wear RFID tags; of the 1,200 attendees, 405 volunteers wore the tags. Face-to-face interactions between these 405 volunteers were collected during 2 days of the conference (3rd and 4th of June 2009). The data were collected from 9 am to 9 pm on the first day and from 8.30 am to 4.30 pm on the second day (periods defined as 'day' in the following text). Contacts were not recorded outside of these time periods (periods defined as 'nights').

### Empirical contact networks

To assess the role of the dynamic nature of the network of contacts in the dynamics of disease spread, we considered a network built on the explicit representation of the dynamic interactions between individuals (referred to as the dynamic network; DYN) at the shortest available temporal resolution (20 seconds) against two benchmark networks that are built on progressively lower amounts of information available on the interactions, referred to as the heterogeneous (HET) and homogenous (HOM) networks, respectively.

Firstly, taking advantage of the full spatial and temporal resolution, DYN considered the empirical sequence of successive contact events collected during the congress. Each contact was identified by the RFID identification numbers of the two individuals involved, and by its starting and ending times. The resulting network was a dynamic object encoding the actual chronology and duration of contacts, therefore preserving heterogeneity in the duration of contacts and the causality constraints between events. The latter is particularly important for disease spread, as it may prevent propagation along certain sequences of interactions that would otherwise be allowed in an aggregated static representation of the contact patterns. For example, if a susceptible individual A interacts first with an infectious individual B and then with a susceptible individual C, disease transmission can occur from B to A and then from A to C. If instead, A meets first C and later B, A can become infected from B, but the propagation from B to A and then to C is no longer possible.

The benchmark networks correspond to coarse-graining of the data on a daily scale. The first one, HET, was produced for each conference day by connecting individuals who came in contact during this conference day, thus aggregating all daily dynamic information in a single snapshot, and weighting each link by the total time the two individuals spent in face-to-face presence during the considered day. Therefore, HET included information on the actual contacts between individuals (who has met whom) and on the total duration of these contacts (how long A was in contact with B during the whole day), but disregarded information about the temporal order of contacts. In the previous example, the transmission from A to C could take place in both situations, representing the different sequences of the events. HET was therefore a daily aggregated network in which contacts were aggregated over a day, but the whole neighborhood structure between individuals was kept. As the conference lasted 2 days, the aggregation procedure produced two such networks, one for each day.

By contrast, the HOM network was constructed for each day by connecting individuals who were in face-to-face contact during the conference day, again aggregating all daily dynamic information in a single snapshot, but weighting each link with equal weight, corresponding to the mean duration of contacts between two individuals who have met each other on the same day in the HET network. The HOM construction may correspond to networks constructed by asking each participant to report with whom they have been in contact during the conference day, and then estimating for how long on average this contact lasted. For each conference day, HET and HOM have exactly the same structure of interactions from a topological point of view, but they differ by the assignments of weights on the links.

### Generation of contact networks on longer timescales

Because we simulated the spread of a realistic infectious disease, which would be characterized by longer timescales than the data collection period, we introduced three different procedures to longitudinally extend the data-driven network, by preserving some of its features. The simplest procedure consisted of repeating the 2-day recordings. This repetition procedure, denoted as REP, was performed both for the dynamic sequence of contacts (DYN) and consistently for the set of daily HET and HOM networks. In this simple procedure, the same contacts were repeated for each attendee for each simulated sequence of 2 days; that is, the assumption was made that the same attendee always met the same set of other attendees, in the same order, and for the same duration. Although this procedure yields a realistic contact pattern for each single day, it uses only empirical data, and thus such a 'deterministic' repetition is rather unrealistic as time goes on. We therefore considered two additional procedures that might improve this limitation.

The first one, random shuffling (RAND-SH), consisted of producing 2-day sequences by randomly reshuffling the participants' identities, as given by their tag IDs. The overall sequence of contacts was preserved, but each contact was set as occurring between different attendees from one 2-day sequence to the next. DYN networks were then constructed as before, taking into account the 20- second temporal resolution, and the HET and HOM networks were obtained by aggregating the data for each day, as explained above. This method results in more realistic contact patterns being obtained, and avoids the unrealistic repetition of interactions between individuals. However, the RAND-SH procedure completely erases any correlations between the contact patterns of an attendee in successive 2-day sequences, which is also unrealistic. Analysis of the empirical contact networks shows that in fact a correlation did exist between the number of contacts of an attendee in the first and second conference days, and also that a fraction of contacts were repeated from one day to the next.

Therefore, we designed a third procedure (constrained shuffling; CONSTR-SH) for the generation of synthetic contact patterns starting from the 2-day sequence, which constrained the reshuffling to preserve the correlations between the attendees' social activity and the same fraction of repeated contacts during successive days (see Additional file 1).

It is important to note that in all cases we preserved the time frame during which data were collected, because no collection occurred outside the conference premises. For this reason, each individual was considered as isolated during the 'night' periods in the DYN network. We therefore also introduced such 'nights' in the HET and HOM networks by 'switching off' the links (that is, considering individuals as isolated) during these periods, thus resembling the circadian pattern encoded by the empirical data.

### Epidemiological model

We considered a simple SEIR epidemic model for the simulation of the infectious-disease spread in the population under study, in which no births, deaths or introduction of new individuals occurred. Individuals were each assigned to one of the following disease states: Susceptible (S), Exposed (E), Infectious (I) or Recovered (R).

The model is individual-based and stochastic. Susceptible individuals may contract the disease with a given rate when in contact with an infectious individual, and enter the exposed disease state when they become infected but are not yet infectious themselves. These exposed individuals become infectious at a rate σ, with σ^-1 ^representing the mean latent period of the disease. Infectious individuals can transmit the disease during their infectious period, whose mean duration is equal to *v*^-1^. After this period, they enter the recovered phase, acquiring permanent immunity to the disease.

To compare simulation results obtained from the three different networks, we needed to adequately define the rate of infection for a given infectious-susceptible pair, depending on the definition of the networks themselves. β was defined as the constant rate of infection from an infected individual to one of their susceptible contacts on the unitary time step *dt *of the process. Given two people, an infectious individual A and a susceptible individual B, who are in contact during the unitary time step, the probability of B becoming infected during this period was given by β*dt*. To obtain the same mean infection probability in the HET and HOM networks over an entire 24-hour period (day and night), the weights on such networks needed to be rescaled by *W_AB_*/Δ*T*, defined as the ratio between the total sum of the duration of all contacts between A and B in a day, and the effective duration of the day (that is, the total time during which the links in the daily networks were considered active, discarding the 'nights'). Therefore, the probability of infection between A and B during the time step *dt *was *βW*_AB _*dt*/ΔT for the HET network, and *β<W> dt*/ΔT for the HOM network (with <W> being the mean weight of the links in the HET network).

We considered two different disease scenarios for the simulations of disease spread on all networks under study. In particular, the following values were assumed for the duration of the mean latency period (σ^-1^), mean infectious period (*v*^-1^) and transmission rate (β): (i) σ^-1 ^= 1 days, *v*^-1 ^= 2 days and β = 3.10^-4^/s (very short incubation and infectious periods); and (ii) σ^-1^= 2 days, *v*^-1 ^= 4 days and β = 15.10^-5^/s (short incubation and infectious periods). These sets of parameter values were chosen to maintain the same value of β/*v*, which is the biologic factor responsible for the rate of increase of cases during the epidemic outbreak, while changing the global timescales of incubation and infectious periods, and assessing the role played by the social factors embedded in the contact patterns. Short incubation and infectious periods were used so as to minimize the consequences of the arbitrariness in the construction procedures of long datasets as described above. Each simulation started with a single randomly chosen infectious individual, with the rest of the population being in the susceptible state.

### Analysis of the empirical contact networks and of the simulation results

To describe the empirical contact networks, we calculated the number of contacts, the mean duration of contacts, the mean degree of a node (defined as the number of distinct individuals encountered by the individual under scrutiny), the mean clustering coefficient (which describes the local cohesiveness), the mean shortest path (defined as the mean number of links to cross to go from one node to another, and the correlation between the properties of the nodes in the aggregated networks of the first and second conference day). For this analysis, we measured the Pearson correlation coefficients between the degree of an individual in the first and second day, and between the time spent in interaction in the first and second day.

The comparison of the epidemic outbreaks in the three networks under study was performed by analyzing several parameters, namely the final size of the epidemic, the number of infectious individuals during the epidemic peak, the time of the peak, and the duration of the epidemic.

Since we aim at assessing the impact on spreading phenomena of the contact patterns, of their dynamic nature, and of the available amount of details on their dynamics we also estimated the reproductive number *R*_0_, defined as the expected number of secondary infections from an initial infected individual in a completely susceptible host population [1]. Several methods can be used to compute *R*_0 _[40,41], possibly yielding different estimates [42] for the same epidemiological parameters. In this study, we computed the value of *R*_0 _as the mean, over different realizations, of the number of secondary cases from the single initial randomly chosen infectious individual. Mean *R*_0 _values and variances were then compared for the three networks (DYN, HET and HOM) and the three data-extension procedures (REP, RAND-SH and CONSTR-SH) under study.

## Results

**Figure 1:**
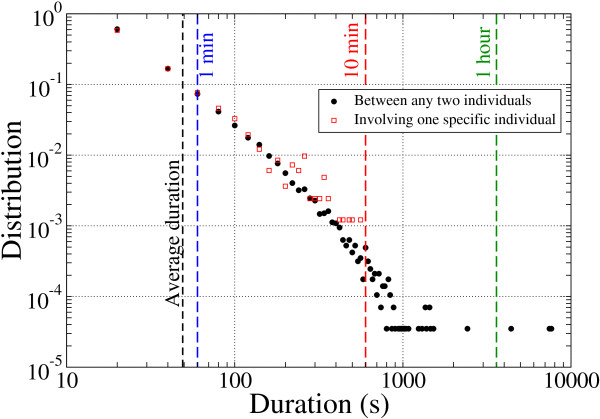
**Distribution of the contact duration between any two people on a log-log scale**. The mean duration was 49 seconds, with SD 112 seconds.

In total, 28,540 face-to-face contacts between 405 attendees at a 2-day conference were recorded, and the probability distribution of the duration of these contacts was plotted (Figure 1). The mean duration was 49 seconds, with large variations (SD 112 seconds), meaning a large number of contacts of brief duration, a few contacts of long duration, and a broad tail, suggesting that no typical contact duration could be defined. Statistical distributions of the number and duration of contacts and of the link weights were similar from one day to the next, although the two daily contact networks were obviously not identical.

In the daily contact networks, the mean degree of a node was close to 30, with a distribution decaying exponentially for large numbers. The mean clustering coefficient was 0.28, much larger than the mean value of 0.07 obtained for a random network of the same size and mean degree. The network was also a small world, with a mean shortest path of 2.2 (snapshots of the network of the first conference day are shown; see Additional File 2).

The link weights, by contrast, had a broad distribution, with a mean cumulated duration of the interaction between two attendees of 2 minutes. The total duration spent in contact by any attendee also had a broad distribution, with a mean of 75 minutes. The Pearson correlation coefficient between the degree of an individual in the first and second day was 0.37, and that between the total time spent in interaction in the first and second day was 0.52. The fraction of repeated contacts in the second day with respect to the first was 12%, and was independent of the degree.

**Figure 2:**
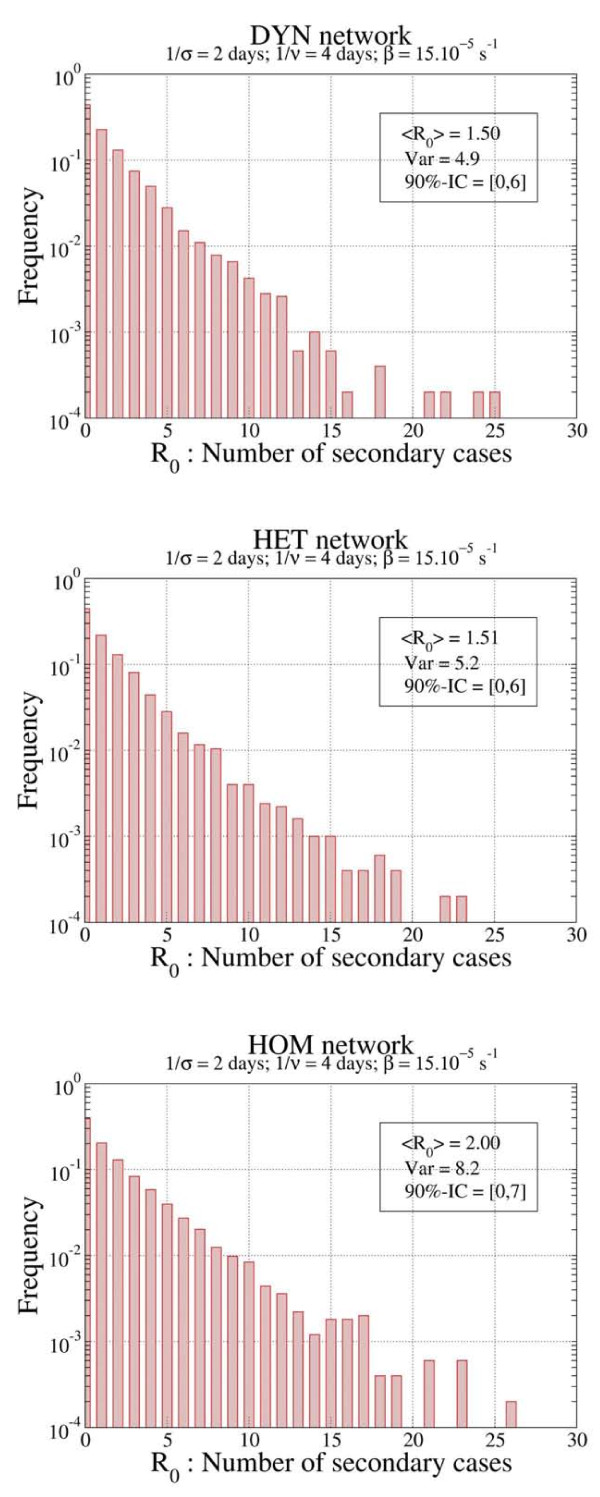
**Distribution of R_0 _for the homogenous (HOM), heterogenous (HET) and dynamic (DYN) networks with the parameters σ^-1 ^= 2 days, *v*^-1 ^= 4 days and β = 15.10^-5^/s, in the repetition (REP) procedure**.

**Figure 3:**
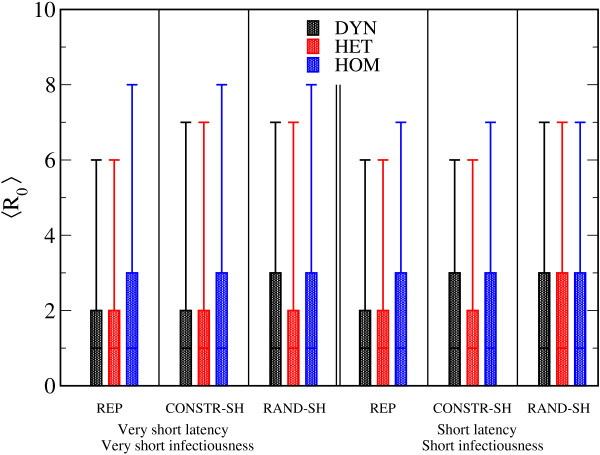
**Boxplots showing the distributions of R_0 _according to the different scenarios and network types**. The bottom and top of the rectangular boxes correspond to the 25th and 75th quantile of the distribution, the horizontal lines to the median, and the ends of the whiskers give the 5th and 95th percentiles. Very short latency, very short infectiousness scenario: σ^-1 ^= 1 days, *v*^-1 ^= 2 days and β = 3.10^-4^/s. Short latency, short infectiousness scenario: σ^-1 ^= 2 days, *v*^-1 ^= 4 days and β = 15.10^-5^/s.

The distributions of *R*_0 _for the three networks using the REP procedure were also plotted (Figure 2). In all cases, the number of secondary cases from the initial seed of the single infectious individual ranged from 0, corresponding to the most probable event of no outbreak, to around 20 to 25 individuals (the mean values and the variances obtained for the estimation of *R*_0_, depending on the scenarios and the network type are shown: Figure 3; see Additional file 3). In all scenarios, higher values of *R*_0_, together with larger variances, were observed in the HOM network compared with the HET and DYN networks.

**Figure 4:**
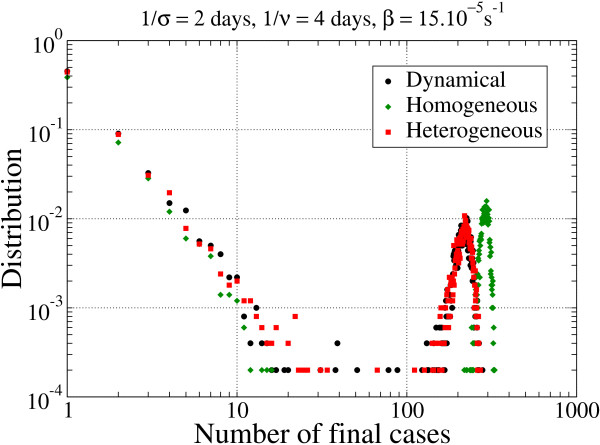
**Distribution of the final number of cases for the three networks with the parameters σ^-1 ^= 2 days, *v*^-1 ^= 4 days and β = 15.10^-5^/s (short latency, short infectiousness), in the repetition (REP) procedure**.

The distribution of the final number of cases for the three networks and the REP data-extension procedure are also shown (Figure 4). In this plot, a high probability of rapid extinction of the pathogen spread was seen, corresponding to a small number of infected individuals. This was slightly smaller in the HOM case compared with the HET and DYN networks. By contrast, when the epidemic started, the final number of cases was high, and it was larger in the HOM network than in the HET and DYN networks. Intermediate cases with limited propagation were rare.

The distribution of the final number of cases for the three networks was analyzed for the various parameters of the SEIR model and for the various extrapolation scenarios (Table 1; see Additional file 4). In all cases, and independently from the procedure adopted for extending the 2-day dataset, the probability of extinction for the HOM network was lower than for the HET and DYN networks. In the case of large outbreaks, the final number of cases was higher in the HOM network than in the HET and DYN networks. Propagation over the HET and DYN networks led to a similar extinction probability and to a similar final number of cases. The final numbers of cases for both disease scenarios (i.e., short and very short latency and infectious periods) were also fairly close.

**Table 1:** Distribution of the final number of cases for the three network types according to the four scenarios (5000 runs, dynamic contact network of 405 participating attendees)

					1 to 10 final cases (AR* ≤ 2.5%)			11 to 40 final cases (2.5% < AR ≤ 10%)			> 40 final cases (AR > 10%)		
					
Scenarios	Parameters	Network^a^	Runs, n	% of run with no secondary cases	% run	Mean cases, n	90% CI	% run	Mean cases, n	90% CI	% run	Mean cases, n	90% CI
REP**^b^**													

Very short latency	σ^-1 ^= 1 days	DYN	5000	47.3	18.2	2.3	1 to 6	0.7	15.9	11 to 22	33.8	208	169 to 242
		
Very short infectiousness	*v*^-1 ^= 2 days	HET	5000	46.4	17.7	2.4	1 to 7	0.8	17.9	11 to 32	35.2	210	171 to 243
		
Transmission rate	β = 3.10^-4^/s	HOM	5000	41.7	11.7	2.2	1 to 6	0.2	16.6	11 to 30	46.3	285	257 to 310

Short latency	σ^-1 ^= 2 days	DYN	5000	45.3	17.0	2.2	1 to 7	0.4	18.3	11 to 38	37.3	214	178 to 246
		
Short infectiousness	*v*^-1 ^= 4 days	HET	5000	44.4	16.4	2.2	1 to 6	0.6	16.8	11 to 27	38.6	216	178 to 248
		
Transmission rate	β = 15.10^-5^/s	HOM	5000	38.7	13;2	2.1	1 to 6	0.1	13.2	11 to 15	48.1	288	262 to 310

RAND-SH**^c^**													

Very short latency	σ^-1 ^= 1 days	DYN	5000	44.8	19.4	2.8	1 to 8	2.2	17.9	11 to 31	33.6	278	223 to 319
		
Very short infectiousness	*v*^-1 ^= 2 days	HET	5000	45.4	18.5	2.6	1 to 7	1.6	17.6	11 to 30	34.5	284	241 to 322
		
Transmission rate	β = 3.10^-4^/s	HOM	5000	39.9	14.3	2.6	1 to 7	0.8	15.7	11 to 28	45.0	324	291 to 350
		
Short latency	σ^-1 ^= 2 days	DYN	5000	40.6	18.6	2.7	1 to 8	1.4	19.2	11 to 31	39.4	297	254 to 331
		
Short infectiousness	*v*^-1 ^= 4 days	HET	5000	39.5	18.0	2.7	1 to 8	1.3	16.7	11 to 30	41.2	300	259 to 333
		
Transmission rate	β = 15.10^-5^/s	HOM	5000	35.9	15.7	2.5	1 to 7	0.9	17.0	11 to 31	47.5	325	293 to 352

CONSTR-SH**^d^**													

Very short latency	σ^-1 ^= 1 days	DYN	5000	45.4	17.7	2.4	1 to 7	1.0	17.0	11 to 28	35.8	240	194 to 278
		
Very short infectiousness	*v*^-1 ^= 2 days	HET	5000	46.8	16.5	2.4	1 to 7	0.8	19.0	11 to 33	35.9	245	202 to 282
		
Transmission rate	β = 3.10^-4^/s	HOM	5000	39.8	13.3	2.3	1 to 6	0.7	15.4	11 to 21	46.2	308	278 to 334
		
Short latency	σ^-1 ^= 2 days	DYN	5000	40.9	18.2	2.3	1 to 6	0.8	16.8	11 to 34	40.2	258	215 to 292
		
Short infectiousness	*v*^-1 ^= 4 days	HET	5000	41.3	16.8	2.3	1 to 7	0.5	14.0	11 to 25	41.4	257	213 to 292
		
Transmission rate	β = 15.10^-5^/s	HOM	5000	35.7	14.8	2.4	1 to 7	0.4	15.2	11 to 21	49.2	314	284 to 339

^a^Networks: DYN = dynamic; HET = heterogenous; HOM = homogenous.

**^b^**Repetition.

**^c^**Random shuffling.

**^d^**Constrained shuffling.

**Figure 5:**
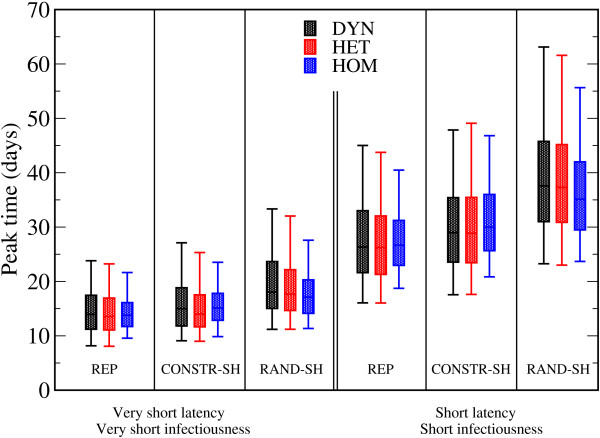
**Boxplots (symbols as in Fig 3.) showing the distributions of the prevalence peak time t_peak _according to the different scenarios and network types**. Only runs with attack rate (AR) > 10% were taken into account. Very short latency, very short infectiousness scenario: σ^-1 ^= 1 days, *v*^-1 ^= 2 days and β = 3.10^-4^/s. Short latency, short infectiousness scenario: σ^-1 ^= 2 days, *v*^-1 ^= 4 days and β = 15.10^-5^/s.

Regarding the peak times of disease spread in the various cases (Figure 5; see Additional file 5), we found that in most cases, the peak of the epidemic was reached first on average for spread within the HOM network. However, the differences between the peak times were small, and even the simulations on the network with the least information gave a good estimate of the peak time obtained when the full information on the contact patterns was included.

**Figure 6:**
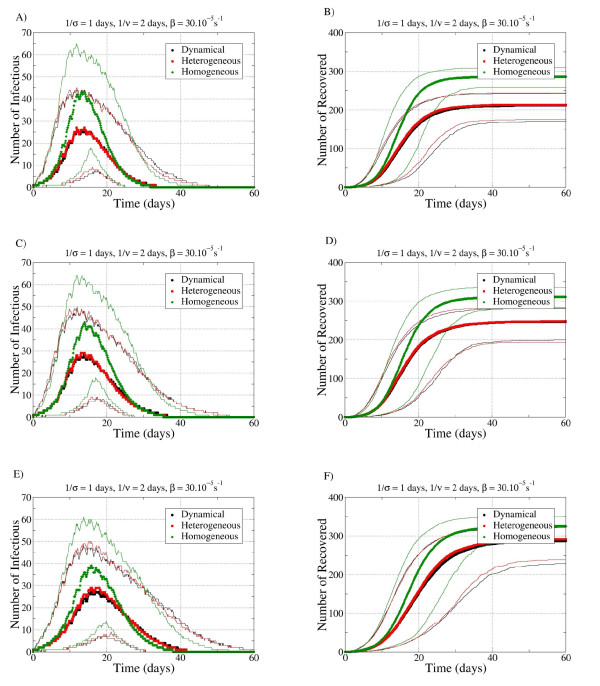
**Temporal evolution of the spreading process for the three networks with the parameters σ^-1 ^= 1 days, *v*^-1 ^= 2 days and β = 3.10^-4^/s (very short latency, very short infectiousness)**. (**A, C, E**) Evolution of the number of infectious individuals; (**B, D, F**) number of recovered. (**A, B**) Repetition (REP) procedure; (**C, D**) to the constrained shuffling (CONSTR-SH) procedure and panels E, F to the random shuffling (RAND-SH) one. Only runs with AR > 10% are taken into account. Symbols represent the median values, and lines represent the fifth and ninety-fifth percentiles of the number of infectious and recovered individuals.

**Figure 7:**
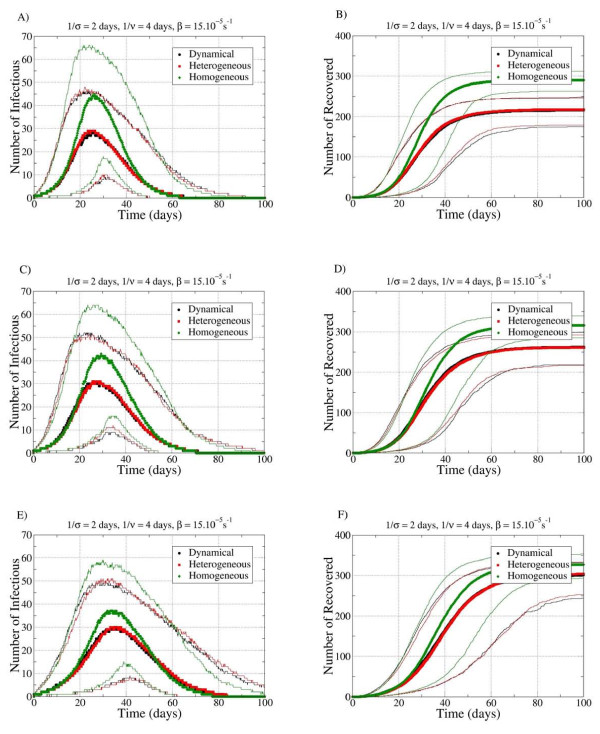
**Distribution of the final number of cases for the three networks with the parameters σ^-1 ^= 2 days, *v*^-1 ^= 4 days and β = 15.10^-5^/s (short latency, short infectiousness) in the repetition (REP) procedure**.

Using the evolution in time of the number of infectious and recovered individuals for the different data-extension procedures and for the two sets of SEIR parameters, the temporal behavior of disease spread was analyzed (Figure 6; Figure 7). Symbols represent the median values, and lines represent the fifth and ninety-fifth percentiles of the number of infectious and recovered individuals. In all cases, disease spread on the HOM network evolved slightly faster and reached a significantly larger number of individuals, compared with the HET and DYN, which had very similar characteristics to each other.

Interesting differences were seen in the results of simulations on datasets extended with different procedures (Figure 5, Figure 6, Figure 7). The spread was slightly slower in the RAND-SH case, but lasted longer, ad consequently the final number of cases R_∞ _was larger. In fact, we systematically found *R*_∞_(REP) <*R*_∞_(CONSTR-SH) <*R*_∞_(RAND-SH), and the more the identities of the tags were shuffled, the more efficient was the spread.

## Discussion

Using a recently developed data collection technique deployed during a 2-day conference involving 405 volunteers, we measured the dynamics of contact (face-to-face) interactions between individuals during such a social event. We used the data to compare the simulated spread of communicable diseases on this dynamic network (DYN) and on two networks, one heterogeneous (HET) and one homogeneous (HOM), obtained by aggregating the dynamic network at two distinct levels of precision. To compensate for the relatively short duration of the observation period (2 days), we designed two different models to construct dynamical contact networks spanning an extended time period during which the spread of an infectious disease could be simulated.

The broad distributions of the various network characteristics reported in this study were consistent with those seen in other contexts [30,36,37]. Our results bear also similarity with those reported previously for interaction networks at conferences [30,36], in which the resulting picture was not characterized by the presence of 'superspreaders', when they were defined in terms of the number of distinct individuals contacted. This was however less clear when the cumulated interaction time was taken into account.

In the three networks, disease extinction occurred as frequently (between 36% and 47%) as large outbreaks (between 34% and 49%). Outbreaks tended to be explosive (attack rate between 51% and 80%), consistently with previous work [4]. A large difference in the process of disease spread was apparent between the HOM network (which did not include any information on the heterogeneity of contact durations nor on the dynamic aspect) and the two other networks; for the HOM network there was a systematically larger number of infected individuals. This result implies that heterogeneity in the contact durations between individuals is associated with a lower spread of transmission, suggesting that a single individual who does not spend their time equally between their contacts effectively reduces the routes of disease spread [12,15]. Disregarding the heterogeneity of contact durations can lead to large differences in the estimated number of cases, suggesting that information on the daily cumulated contact time between individuals gives crucial information for correct modeling of disease spread. Interestingly, however, the peak time was only slightly changed in the HOM network, showing that even rather limited information can yield good estimates of the epidemic timescales.

The comparison between disease spread in the HET and DYN networks provides insights into whether temporal constraints due to the precise sequence of the contacts might affect the propagation of disease. Given two individuals, the overall expected probability of a transmission occurring during the interval ΔT is the same in both cases (that is, *βW*_AB_), so the only difference is that the contact is not continuously present in the DYN network, but it may be intermittent and repeated only during the actual recorded contacts. This introduces time constraints on the paths that the infectious agent can follow between individuals in the DYN network, which may slow down disease spread on the DYN network compared with the HET network. However, this slowing down of infection and the differences in the final number of cases between the HET and DYN networks were too small to be relevant for the simulations investigated here. The similarity between the spreading behaviors in the HET and DYN networks was independent of the different procedures used to extend the initial 2-day dataset. These procedures created successive artificial 'days' which differed from each other by various amounts, that is, with a different level of repetition of contacts from one day to the next. The robustness of the comparison between HET and DYN therefore indicates that the observed similarity between the spreading on the HET and DYN networks is due to the discrepancy between the timescales considered for propagation (of the order of days), and the temporal resolution and the contact durations (of 20 seconds and of the order of minutes up to a few hours, respectively). The total time spent in contact by each pair of individuals was in this context sufficient to describe precisely the propagation pattern, as shown by the peak time and the final number of cases. Therefore, for the simulation of diseases such as those considered in this study, contact information at a daily resolution might be enough to characterize disease spread, and the precise order of the sequence of contacts might not be needed. However, this would not be the case for extremely fast-spreading processes, as shown in previous work [36]. This implies that there is a crossover between the two regimens, which will be the subject of future investigations.

Finally, the difference between the results obtained for the different procedures REP, RAND-SH and CONSTR-SH shows the importance of knowledge of the respective fractions of repeated and new contacts between successive days [8,12,43]. Repeated encounters favor propagation, so that the REP procedure led to an initially faster spread, but contacts between different individuals from one day to the next favor propagation across the network, so that the RAND-SH procedure led in the end to a larger attack rate.

Compared with other approaches [11,26,27], the data collection method used in this study makes it possible to gather information on actual face-to-face contacts, with high temporal and spatial resolution [28,30,36]. It allows access to the precise durations, time and order of the successive contacts between individuals, fully representing the corresponding heterogeneity and the causality constraints in the chain of transmission.

## Limitations

Unsupervised data-collection systems based on RFID infrastructures, such as the one presented here [28,30,37] carry some caveats that need to be discussed. First, individuals are not followed outside of the zone covered by RFID readers, so that contacts between participants that occur during the day outside of the area covered by the RFID readers are not monitored. This results in an underestimation of the number of contacts, and therefore of the possibilities for disease spread. Moreover, in this study, the periods of 'nights' represented a proportion of 56% of the 24-hour period, during which individuals were assumed to be isolated. This may artificially increase the probability of extinction if the contagiousness period of an infected individual ends during these periods, precluding further transmission. This issue may be solved by upcoming technological improvements that will allow operation of the RFID sensing layer in a fully distributed fashion with on-board storage on the devices themselves; that is, such RFID tags will register and store contacts even if they are not close to RFID readers.

Another issue, well known in the field of social networks, is due to the partial sampling of the population. Of the 1,200 attendees at this conference, 405 (34%) participated in the data collection. Consequently, only these attendees were taken into account in the model of disease spread, whereas they were in fact also in contact with the non-participating attendees. Previous investigation [30] has shown that for a wide variety of real-world deployments of the RFID proximity-sensing platform used in this study, the behavior of the statistical distributions of quantities such as contact durations is not altered by unbiased sampling of individuals. However, paths of disease spread between sampled attendees that also involved unsampled attendees may have existed, but were not taken into account. This effect may lead to an underestimation of disease spread, and future work will focus on quantification of such possible biases, for instance through bootstrapping procedures. In addition, it is possible that the volunteering participants themselves introduced a systematic bias into the sampled population concerning their interaction behavior, as they self-selected to participate to the experiment. However, assessment of this effect would require independent data sources for monitoring unsampled individuals, inevitably limiting the size of populations and settings because of logistics constraints. Although interesting for the understanding of social behavior, such a study would need to be specifically designed and tailored to the research question, thus going beyond the aim of the present study. Another interesting perspective would be to compare and integrate the results of unsupervised contact measurements with the results of simultaneously performed survey- or diary-based inquiries.

Finally, the limited period (2 days) of data collection made it necessary to generate artificially longer datasets by different procedures in order to model the spread of pathogens on realistic timescales. Deployment of the measuring infrastructure on much longer timescales is planned so as to validate such generation procedures and to measure their effect.

## Conclusions

Despite the limitations described above, the present study emphasizes the effects of contact heterogeneity on the dynamics of communicable diseases. On the one hand, the small differences between simulated spread on both the HET and DYN networks shows that taking into account the very detailed actual time ordering of the contacts between individuals, with a time resolution of minutes, does not seem to be essential to describe disease spread on a timescale of several days or weeks. On the other hand, the large differences in disease spread in the HOM network emphasize the need to include detailed information about the heterogeneity of contact duration (compared with an assumption of homogeneity) to model disease spread, as also found previously [12,13] for simulations of disease spread dynamics based on diary-based survey data. Results from the different procedures for data extension also showed how the rate of new contacts is a very important parameter [8,12,43]. Overall, the combined comparison of the spreading processes simulated on the HET, DYN and HOM networks and using the different data-extension procedures gave an important assessment of the level of detail concerning the contact patterns of individuals that is needed to inform modeling frameworks of epidemic spread.

In this context, a data collection infrastructure such as the one used in this study seems to be very effective, as it gives access to the level of information needed, and also allows the simulation of very fast-spreading processes characterized by timescales comparable with those intrinsic to social dynamics, where even the precise ordering of contact events becomes crucial. These measurements should be also extended to other contexts in which individuals interact closely in different ways, such as workplaces, schools or hospitals [44,45]. More experimental work is needed to collect data over longer time periods, and in particular to understand better how datasets limited in time can be artificially extended to yield realistic datasets, on various samples of individuals and in various locations. The results of these approaches could be helpful to anticipate the effect of preventive measures, and contribute to decisions about the best strategies to control the spread of known or emerging infections.

## Competing interests

The authors declare that they have no competing interests.

## Authors' contributions

JS, NV, AB, CC, VC, LI, CR, JFP, WVdB and PV conceived of and designed the experiments; NV, AB, CC, CR, JFP, NK, WVdB and PV performed the data collection; JS, NV, AB, CC, VC, LI and JFP analyzed the data; and JS, NV, AB, CC, VC, LI, JFP and PV wrote the paper. All authors read and approved the final manuscript.

## Pre-publication history

The pre-publication history for this paper can be accessed here:

http://www.biomedcentral.com/1741-7015/9/87/prepub

## Supplementary Material

Additional file 1 **Supporting text**. Description of the data-extension procedure CONSTR-SH (constrained shuffling_. Click here for file

Additional file 2 **Supplementary figures 1-3**. Snapshots of the contact graph between the 405 attendees for the first conference day. Click here for file

Additional file 3 **Supplementary table 1**. Mean values, variances and 90% CI of R_0 _according to the different scenarios and network types. Click here for file

Additional file 4 **Supplementary figure 4**. Box plots showing the distributions of the number of final cases when the final attack rate is larger than 10%, according to the different scenarios and network types. Click here for file

Additional file 5 **Supplementary table 2**. Mean values, variances and 90% CI of the prevalence peak time t_peak _according to the different scenarios and network types. Click here for file

## References

[B1] Anderson RM May RM Infectious Diseases of Humans: dynamics and control 1991 Oxford University Press

[B2] Liljeros F Edling CR Amaral LA Stanley HE Aberg Y The web of human sexual contacts Nature 2001 411 907 8 10.1038/35082140 11418846

[B3] Lloyd AL May RM Epidemiology. How viruses spread among computers and people Science 2001 292 1316 7 10.1126/science.1061076 11360990

[B4] Lloyd-Smith JO Schreiber SJ Kopp PE Getz WM Superspreading and the effect of individual variation on disease emergence Nature 2005 438 355 9 10.1038/nature04153 16292310 PMC7094981

[B5] Pastor-Satorras R Vespignani A Epidemic spreading in scale-free networks Phys Rev Lett 2001 86 3200 3 10.1103/PhysRevLett.86.3200 11290142

[B6] Eames KT Modelling disease spread through random and regular contacts in clustered populations Theor Popul Biol 2008 73 104 11 10.1016/j.tpb.2007.09.007 18006032

[B7] Keeling MJ The effects of local spatial structure on epidemiological invasions Proc Biol Sci 1999 266 859 67 10.1098/rspb.1999.0716 10343409 PMC1689913

[B8] Smieszek T Fiebig L Scholz RW Models of epidemics: when contact repetition and clustering should be included Theor Biol Med Model 2009 6 11 10.1186/1742-4682-6-11 19563624 PMC2709892

[B9] Szendroi B Csanyi G Polynomial epidemics and clustering in contact networks Proc Biol Sci 2004 271 Suppl 5 S364 6 15504019 10.1098/rsbl.2004.0188 PMC1810049

[B10] Zaric GS Random vs. nonrandom mixing in network epidemic models Health Care Manag Sci 2002 5 147 55 10.1023/A:1014489218178 11993749

[B11] Mossong J Hens N Jit M Beutels P Auranen K Mikolajczyk R Massari M Salmaso S Tomba GS Wallinga J Heijne J Sadkowska-Todys M Rosinska M Edmunds WJ Social contacts and mixing patterns relevant to the spread of infectious diseases PLoS Med 2008 5 e74 10.1371/journal.pmed.0050074 18366252 PMC2270306

[B12] Read JM Eames KT Edmunds WJ Dynamic social networks and the implications for the spread of infectious disease J R Soc Interface 2008 5 1001 7 10.1098/rsif.2008.0013 18319209 PMC2607433

[B13] Smieszek T A mechanistic model of infection: why duration and intensity of contacts should be included in models of disease spread Theor Biol Med Model 2009 6 25 10.1186/1742-4682-6-25 19919678 PMC2780993

[B14] Balcan D Colizza V Gonçalves B Hu H Ramasco JJ Vespignani A Multiscale mobility networks and the spatial spreading of infectious diseases Proc Natl Acad Sci USA 2009 106 21484 9 10.1073/pnas.0906910106 20018697 PMC2793313

[B15] Colizza V Barrat A Barthélemy M Vespignani A The role of the airline transportation network in the prediction and predictability of global epidemics Proc Natl Acad Sci USA 2006 103 2015 20 10.1073/pnas.0510525103 16461461 PMC1413717

[B16] Eubank S Guclu H Kumar VS Marathe MV Srinivasan A Toroczkai Z Wang N Modelling disease outbreaks in realistic urban social networks Nature 2004 429 180 4 10.1038/nature02541 15141212

[B17] Ferguson NM Cummings DA Fraser C Cajka JC Cooley PC Burke DS Strategies for mitigating an influenza pandemic Nature 2006 442 448 52 10.1038/nature04795 16642006 PMC7095311

[B18] Germann TC Kadau K Longini IM Jr Macken CA Mitigation strategies for pandemic influenza in the United Statpluriel scénario anglaises Proc Natl Acad Sci USA 2006 103 5935 40 10.1073/pnas.0601266103 16585506 PMC1458676

[B19] Hufnagel L Brockmann D Geisel T Forecast and control of epidemics in a globalized world Proc Natl Acad Sci USA 2004 101 15124 9 10.1073/pnas.0308344101 15477600 PMC524041

[B20] Longini IM Jr Nizam A Xu S Ungchusak K Hanshaoworakul W Cummings DA Halloran ME Containing pandemic influenza at the source Science 2005 309 1083 7 10.1126/science.1115717 16079251

[B21] Merler S Ajelli M The role of population heterogeneity and human mobility in the spread of pandemic influenza Proc Biol Sci 2009 277 557 65 19864279 10.1098/rspb.2009.1605 PMC2842687

[B22] Riley S Large-scale spatial-transmission models of infectious disease Science 2007 316 1298 301 10.1126/science.1134695 17540894

[B23] Rvachev LA Longini IM Jr A mathematical model for the global spread of influenza Math Biosciences 1985 75 3 22 10.1016/0025-5564(85)90064-1

[B24] Beutels P Shkedy Z Aerts M Van Damme P Social mixing patterns for transmission models of close contact infections: exploring self-evaluation and diary-based data collection through a web-based interface Epidemiol Infect 2006 134 1158 66 10.1017/S0950268806006418 16707031 PMC2870524

[B25] Edmunds WJ O'Callaghan CJ Nokes DJ Who mixes with whom? A method to determine the contact patterns of adults that may lead to the spread of airborne infections Proc Biol Sci 1997 264 949 57 10.1098/rspb.1997.0131 9263464 PMC1688546

[B26] Wallinga J Teunis P Kretzschmar M Using data on social contacts to estimate age-specific transmission parameters for respiratory-spread infectious agents Am J Epidemiol 2006 164 936 44 10.1093/aje/kwj317 16968863

[B27] Zagheni E Billari FC Manfredi P Melegaro A Mossong J Edmunds WJ Using time-use data to parameterize models for the spread of close-contact infectious diseases Am J Epidemiol 2008 168 1082 90 10.1093/aje/kwn220 18801889

[B28] The SocioPatterns project http://www.sociopatterns.org/

[B29] Brockmann D Hufnagel L Geisel T The scaling laws of human travel Nature 2006 439 462 5 10.1038/nature04292 16437114

[B30] Cattuto C Van den Broeck W Barrat A Colizza V Pinton JF Vespignani A Dynamics of person-to-person interactions from distributed RFID sensor networks PloS One 2010 5 e11596 10.1371/journal.pone.0011596 20657651 PMC2904704

[B31] Kossinets G Watts DJ Empirical analysis of an evolving social network Science 2006 311 88 90 10.1126/science.1116869 16400149

[B32] O'Neill E Kostakos V Kindberg T Fatah gen. Schiek A Penn A Instrumenting the city: developing methods for observing and understanding the digital cityscape Lecture Notes in Computer Science 2006 4206 315 22 10.1007/11853565_19

[B33] Onnela JP Saramäki J Hyvönen J Szabó G Lazer D Kaski K Kertész J Barabási AL Structure and tie strengths in mobile communication networks Proc Natl Acad Sci USA 2007 104 7332 6 10.1073/pnas.0610245104 17456605 PMC1863470

[B34] Pentland A The Global Information Technology Report 2008-2009 2009

[B35] Watts DJ A twenty-first century science Nature 2007 445 489 10.1038/445489a 17268455

[B36] Isella L Stehlé J Barrat A Cattuto C Pinton JF Van den Broeck W What's in a crowd? Analysis of face-to-face behavioral networks J Theor Biol 2010 271 166 180 10.1016/j.jtbi.2010.11.033 21130777

[B37] Salathé M Kazandjieva M Lee JW Levis P Feldman MW Jones JH A high-resolution human contact network for infectious disease transmission Proc Natl Acad Sci (USA) 2010 107 22020 22025 10.1073/pnas.1009094108 PMC3009790 21149721

[B38] Lazer D Pentland A Adamic L Aral S Barabasi AL Brewer D Christakis N Contractor N Fowler J Gutmann M Jebara T King G Macy M Roy D Van Alstyne M Social science. Computational social science Science 2009 323 721 3 10.1126/science.1167742 19197046 PMC2745217

[B39] Barrat A Barthélemy M Vespignani A Dynamical processes on complex networks 2008 Cambridge University Press

[B40] Diekmann O Heersterbeek J Metz J On the definition and the computation of the basic reproduction number ratio R0 in models for infectious diseases in heterogeneous populations J Math Biol 1990 28 365 82 2117040 10.1007/BF00178324

[B41] Heffernan JM Smith RJ Wahl LM Perspectives on the basic reproductive ratio J R Soc Interface 2005 2 281 93 10.1098/rsif.2005.0042 16849186 PMC1578275

[B42] Breban R Vardavas R Blower S Theory versus data: how to calculate R0? PloS One 2007 2 e282 10.1371/journal.pone.0000282 17356693 PMC1804098

[B43] Smieszek T Flebig L Scholz RW Models of epidemics: when contact repetition and clustering should be included Theoretical Biology and Medical Modelling 2009 6 11 10.1186/1742-4682-6-11 19563624 PMC2709892

[B44] Polgreen PM Tassier TL Pemmaraju SV Segre AM Prioritizing healthcare worker vaccinations on the basis of social network analysis Infect Control Hosp Epidemiol 2010 31 893 900 10.1086/655466 20649412 PMC3024853

[B45] Isella L Romano M Barrat A Cattuto C Colizza V Van den Broeck W Gesualdo F Pandolfi E Ravà L Rizzo C Tozzi AE Close encounters in a pediatric ward: measuring face-to-face proximity and mixing patterns with wearable sensors PLoS One 2011 6 e17144 10.1371/journal.pone.0017144 21386902 PMC3046133

