# Supplementary material for: Simulation of an SEIR infectious disease model on the dynamic contact network of conference attendees
Source: BMC Med. 2011 Jul 19;9:87. doi: 10.1186/1741-7015-9-87 (PMC3162551; doi:10.1186/1741-7015-9-87)
Supplement: Additional file 1 — Supporting text. Description of the data-extension procedure CONSTR-SH (constrained shuffling_. [file 1741-7015-9-87-S1.PDF]

# Simulation of a SEIR infectious disease model on the dynamic contact network of conference attendees

## Additional file 1 – Supporting text.

Juliette Stehlé[1], Nicolas Voirin[2,3§], Alain Barrat[1,4], Ciro Cattuto[4], Vittoria Colizza[5,6,7], Lorenzo Isella[4], Corinne Régis[3], Jean-François Pinton[8], Nagham Khanafer[2,3], Wouter Van den Broeck[4] and Philippe Vanhems[2,3]

[1]Centre de Physique Théorique de Marseille, CNRS UMR 6207, Marseille, France

[2]Hospices Civils de Lyon, Hôpital Edouard Herriot, Service d'Hygiène, Epidémiologie et Prévention, Lyon, France

[3]Université de Lyon; université Lyon 1; CNRS UMR 5558, laboratoire de Biométrie et de Biologie Evolutive, Equipe Epidémiologie et Santé Publique, Lyon, France

[4]Data Science Laboratory, Institute for Scientific Interchange (ISI) Foundation, Torino, Italy

[5] INSERM, U707, Paris F-75012, France

[6] UPMC Université Paris 06, Faculté de Médecine Pierre et Marie Curie, UMR S 707, Paris F75012, France

[7]Computational Epidemiology Laboratory, Institute for Scientific Interchange (ISI) Foundation, Torino, Italy

[8]Laboratoire de Physique de l'Ecole Normale Supérieure de Lyon, CNRS UMR 5672, Lyon, France

[§]Corresponding author

**Description of the data extension procedure 'CONSTR-SH'.**

The data describes a list of contact events between pairs of individuals. Upon reshuffling of two tag identities, for instance of tags $i$ and $j$, an artificial data set is generated such that each time the tag $i$ was in contact with another tag, say with $k$, from time $t_0$ to time $t_1$, in the real data, the contact is replaced by a contact between $j$ and $k$ between times $t_0$ and $t_1$.

As explained in the main text, the empirical data set allows constructing daily aggregated contact networks. Let us denote by $f_{emp}$ the observed average fraction of repeated contacts from one day to the next: for each individual $i$, one considers the set $V(i,1)=\{j_1,j_2,...\}$ of individuals with whom $i$ has had a contact on day 1, and $V(i,2)=\{k_1,k_2,...\}$ with whom he or she has had a contact on day 1. The fraction $f_{emp}$ is then the average over all individuals of the ratio between the size of the intersection of $V(i,1)$ and $V(i,2)$, and the size of $V(i,1)$. If $f_{emp} = 0$, it means that $i$ has encountered only new individuals during the second day and if $f_{emp} = 1$, it means that $i$ has encountered exactly the same set of participants in both days.

For each reshuffling of the tags, we can aggregate the reshuffled contact data on a daily scale and create the reshuffled daily contact networks. We then compute the average fraction $f$ of repeated contacts between the empirical and the reshuffled daily aggregated networks. By constraining $f$ to be close to $f_{emp}$, we construct reshuffled contact sequences that conserve a realistic amount of correlations between the sets of individuals encountered from one day to the next in the artificial data set.

We proceed by the following steps:

1. Choose two tag Ids at random
2. Exchange their identities, as described above
3. Compute $f$ and $(f - f_{emp})^2$
4. Accept the exchange with a probability decreasing with $b\ (f - f_{emp})^2$, where $b$ is a parameter
5. Go back to step 1.

By tuning and increasing slowly the parameter $b$, it is then possible to produce reshufflings which have very low values of $(f - f_{emp})^2$, and thus reproduce the empirical correlations between the successive daily networks.
