# Supplementary material for: Simulation of an SEIR infectious disease model on the dynamic contact network of conference attendees
Source: BMC Med. 2011 Jul 19;9:87. doi: 10.1186/1741-7015-9-87 (PMC3162551; doi:10.1186/1741-7015-9-87)

Simulation of a SEIR infectious disease model on the dynamic contact network of conference attendees

Additional file 2 – Supplementary figures 1-3

Juliette Stehlé[1], Nicolas Voirin[2,3§], Alain Barrat[1,4], Ciro Cattuto[4], Vittoria Colizza[5,6,7], Lorenzo Isella[4], Corinne Régis[3], Jean-François Pinton[8], Nagham Khanafer[2,3], Wouter Van den Broeck[4] and Philippe Vanhems[2,3]

[1]Centre de Physique Théorique de Marseille, CNRS UMR 6207, Marseille, France

[2]Hospices Civils de Lyon, Hôpital Edouard Herriot, Service d'Hygiène, Epidémiologie et Prévention, Lyon, France

[3]Université de Lyon; université Lyon 1; CNRS UMR 5558, laboratoire de Biométrie et de Biologie Evolutive, Equipe Epidémiologie et Santé Publique, Lyon, France

[4]Data Science Laboratory, Institute for Scientific Interchange (ISI) Foundation, Torino, Italy

[5] INSERM, U707, Paris F-75012, France

[6] UPMC Université Paris 06, Faculté de Médecine Pierre et Marie Curie, UMR S 707, Paris F75012, France

[7]Computational Epidemiology Laboratory, Institute for Scientific Interchange (ISI) Foundation, Torino, Italy

[8]Laboratoire de Physique de l'Ecole Normale Supérieure de Lyon, CNRS UMR 5672, Lyon, France

[§]Corresponding author

Supplementary figure 1 – Snapshot of the contact graph between the 405 attendees for the first conference day. Each node represents an attendee, and a link between two nodes corresponds to the fact that at least one contact event has been registered between the corresponding attendees.

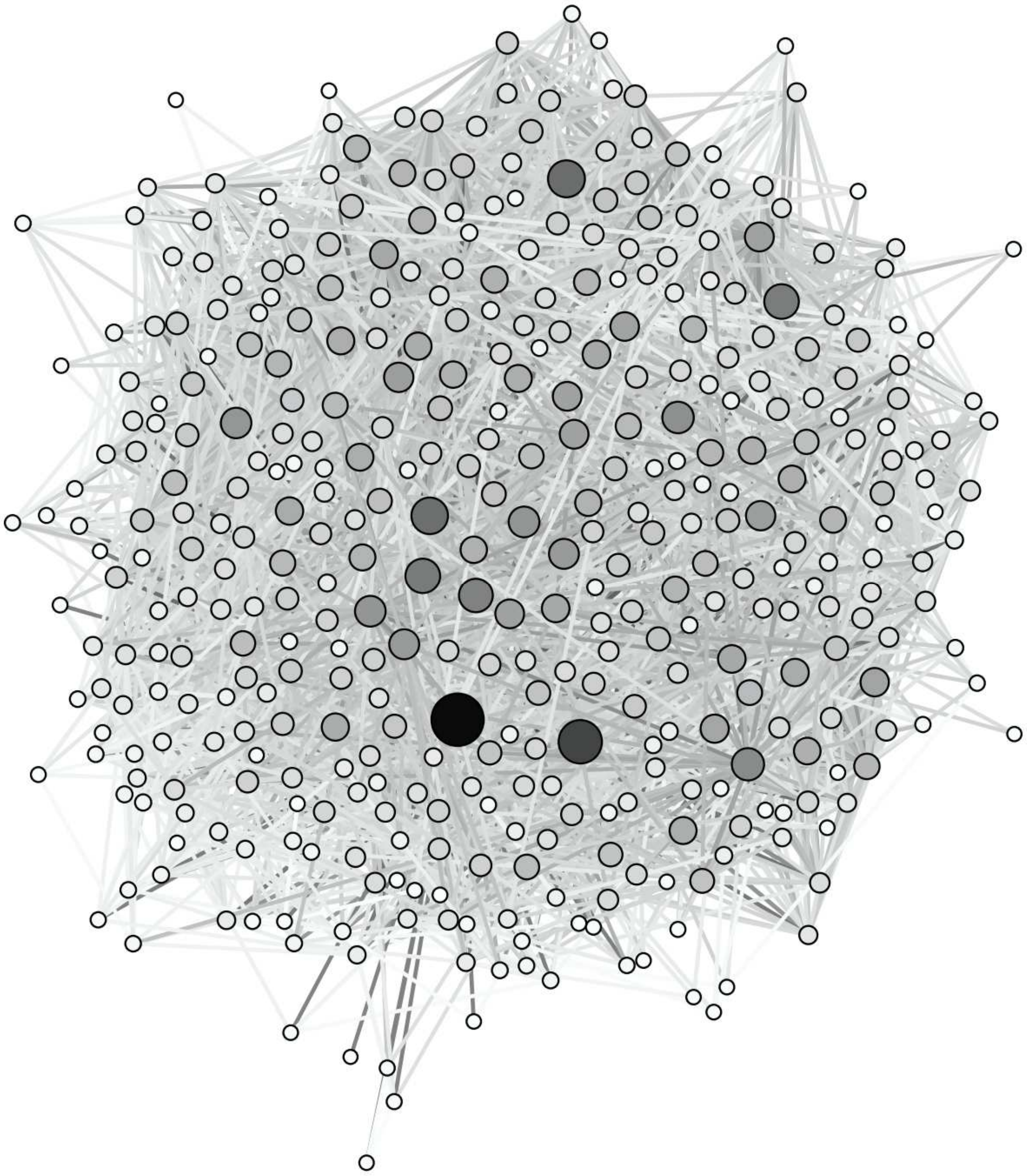

Supplementary figure 2 – Same as supplementary figure 1, in which only links corresponding to a cumulated time spent in proximity of at least 1mn have been kept.

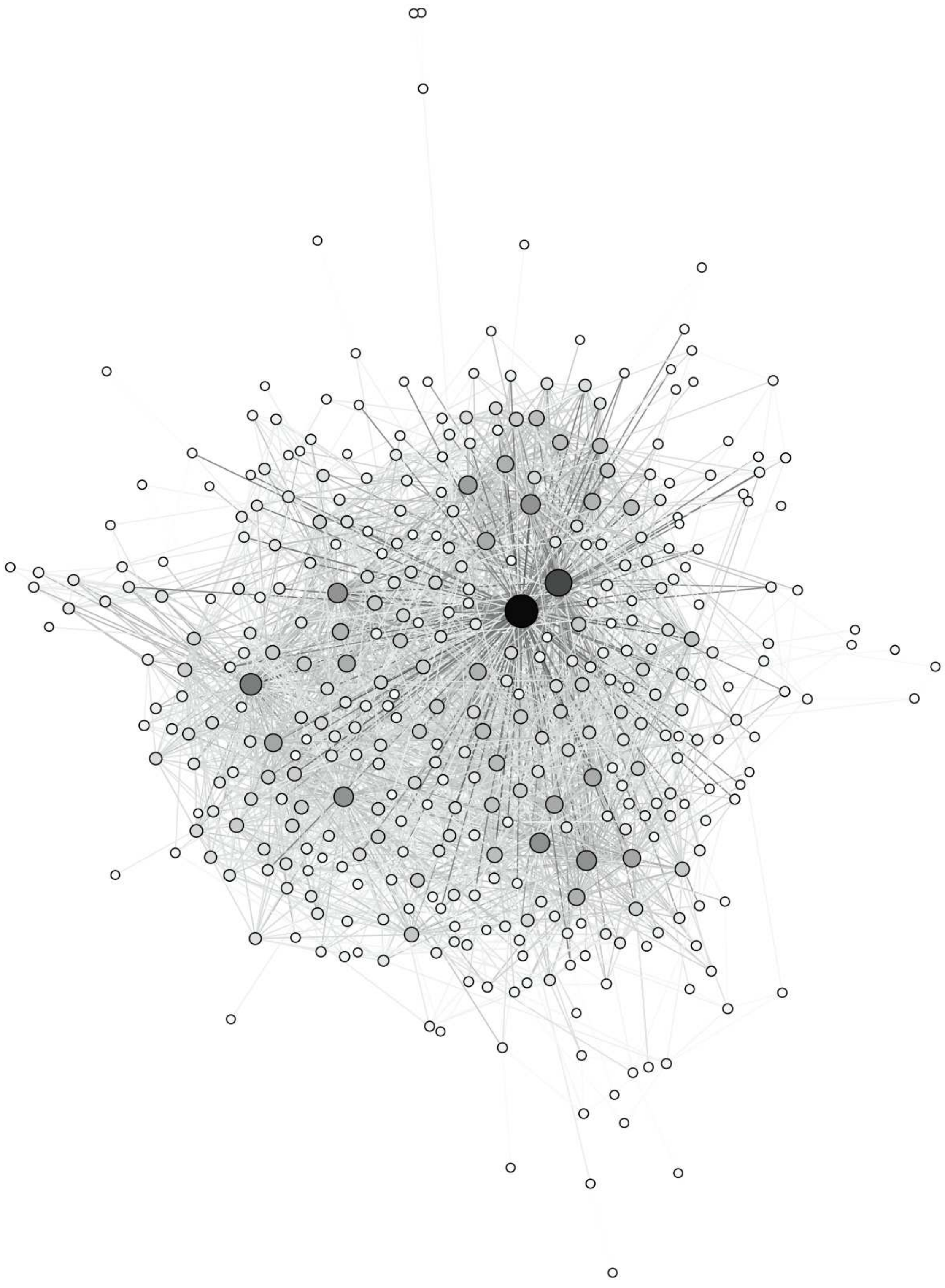

Supplementary figure 3 – Same as supplementary figure 1, in which only links corresponding to a cumulated time spent in proximity of at least 2mn have been kept.

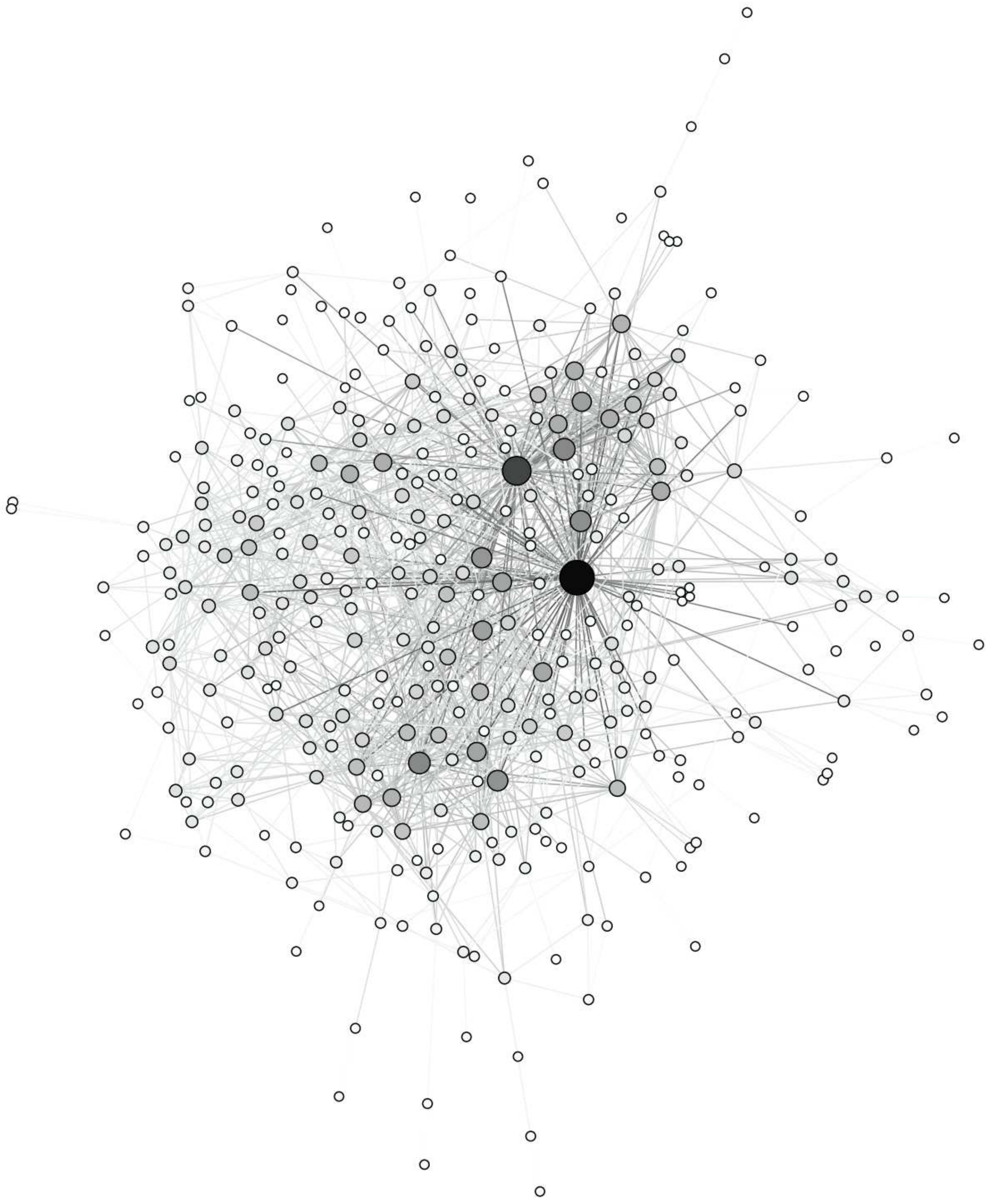

Supplement: Additional file 2 — Supplementary figures 1-3. Snapshots of the contact graph between the 405 attendees for the first conference day. [file 1741-7015-9-87-S2.PDF]
