# Supplementary material for: Simulation of an SEIR infectious disease model on the dynamic contact network of conference attendees
Source: BMC Med. 2011 Jul 19;9:87. doi: 10.1186/1741-7015-9-87 (PMC3162551; doi:10.1186/1741-7015-9-87)
Supplement: Additional file 3 — Supplementary table 1. Mean values, variances and 90% CI of R0 according to the different scenarios and network types. [file 1741-7015-9-87-S3.PDF]

Simulation of a SEIR infectious disease model on the dynamic contact network of conference attendees

Additional file 3 – Supplementary table 1

Juliette Stehlé[1], Nicolas Voirin[2,3§], Alain Barrat[1,4], Ciro Cattuto[4], Vittoria Colizza[5,6,7], Lorenzo Isella[4], Corinne Régis[3], Jean-François Pinton[8], Nagham Khanafer[2,3], Wouter Van den Broeck[4] and Philippe Vanhems[2,3]

[1]Centre de Physique Théorique de Marseille, CNRS UMR 6207, Marseille, France

[2]Hospices Civils de Lyon, Hôpital Edouard Herriot, Service d'Hygiène, Epidémiologie et Prévention, Lyon, France

[3]Université de Lyon; université Lyon 1; CNRS UMR 5558, laboratoire de Biométrie et de Biologie Evolutive, Equipe Epidémiologie et Santé Publique, Lyon, France

[4]Data Science Laboratory, Institute for Scientific Interchange (ISI) Foundation, Torino, Italy

[5] INSERM, U707, Paris F-75012, France

[6] UPMC Université Paris 06, Faculté de Médecine Pierre et Marie Curie, UMR S 707, Paris F75012, France

[7]Computational Epidemiology Laboratory, Institute for Scientific Interchange (ISI) Foundation, Torino, Italy

[8]Laboratoire de Physique de l'Ecole Normale Supérieure de Lyon, CNRS UMR 5672, Lyon, France

[§]Corresponding author

Supplementary table 1 – Average values, variances and 90% confidence interval (90% CI) of R_0 according to the different scenarios and network types.

**Scenarios**	**Parameters**	**Network**	**Number of runs**	**<R_0>**	**Variance**	90% CI
Very short latency<br>Very short infectiousness<br>REP	$1/\sigma = 1$ days<br>$1/\nu = 2$ days<br>$\beta = 3.10^{-4}\ s^{-1}$	DYN	5000	**1.55**	**6.0**	**[0,6]**
		HET	5000	**1.46**	**5.0**	**[0,6]**
		HOM	5000	**1.96**	**7.8**	**[0,8]**
Short latency<br>Short infectiousness<br>REP	$1/\sigma = 2$ days<br>$1/\nu = 4$ days<br>$\beta = 15.10^{-5}\ s^{-1}$	DYN	5000	**1.50**	**5.0**	**[0,6]**
		HET	5000	**1.47**	**4.7**	**[0,5]**
		HOM	5000	**1.93**	**7.7**	**[0,7]**
Very short latency<br>Very short infectiousness<br>RAND-SH	$1/\sigma = 1$ days<br>$1/\nu = 2$ days<br>$\beta = 3.10^{-4}\ s^{-1}$	DYN	5000	**1.99**	**6.9**	**[0,7]**
		HET	5000	**1.70**	**5.9**	**[0,7]**
		HOM	5000	**2.09**	**7.6**	**[0,8]**
Short latency<br>Short infectiousness<br>RAND-SH	$1/\sigma = 2$ days<br>$1/\nu = 4$ days<br>$\beta = 15.10^{-5}\ s^{-1}$	DYN	5000	**1.94**	**6.0**	**[0,7]**
		HET	5000	**1.82**	**6.0**	**[0,7]**
		HOM	5000	**2.03**	**6.5**	**[0,7]**
Very short latency<br>Very short infectiousness<br>CONSTR-SH	$1/\sigma = 1$ days<br>$1/\nu = 2$ days<br>$\beta = 3.10^{-4}\ s^{-1}$	DYN	5000	**1.78**	**7.1**	**[0,7]**
		HET	5000	**1.71**	**6.5**	**[0,7]**
		HOM	5000	**2.09**	**8.2**	**[0,8]**
Short latency<br>Short infectiousness<br>CONSTR-SH	$1/\sigma = 2$ days<br>$1/\nu = 4$ days<br>$\beta = 15.10^{-5}\ s^{-1}$	DYN	5000	**1.79**	**6.3**	**[0,6]**
		HET	5000	**1.72**	**6.3**	**[0,6]**
		HOM	5000	**1.98**	**6.7**	**[0,7]**
