# Supplementary material for: Simulation of an SEIR infectious disease model on the dynamic contact network of conference attendees
Source: BMC Med. 2011 Jul 19;9:87. doi: 10.1186/1741-7015-9-87 (PMC3162551; doi:10.1186/1741-7015-9-87)

Simulation of a SEIR infectious disease model on the dynamic contact network of conference attendees

Additional file 4 – Supplementary figure 4

Juliette Stehlé[1], Nicolas Voirin[2,3§], Alain Barrat[1,4], Ciro Cattuto[4], Vittoria Colizza[5,6,7], Lorenzo Isella[4], Corinne Régis[3], Jean-François Pinton[8], Nagham Khanafer[2,3], Wouter Van den Broeck[4] and Philippe Vanhems[2,3]

[1]Centre de Physique Théorique de Marseille, CNRS UMR 6207, Marseille, France

[2]Hospices Civils de Lyon, Hôpital Edouard Herriot, Service d'Hygiène, Epidémiologie et Prévention, Lyon, France

[3]Université de Lyon; université Lyon 1; CNRS UMR 5558, laboratoire de Biométrie et de Biologie Evolutive, Equipe Epidémiologie et Santé Publique, Lyon, France

[4]Data Science Laboratory, Institute for Scientific Interchange (ISI) Foundation, Torino, Italy

[5] INSERM, U707, Paris F-75012, France

[6] UPMC Université Paris 06, Faculté de Médecine Pierre et Marie Curie, UMR S 707, Paris F75012, France

[7]Computational Epidemiology Laboratory, Institute for Scientific Interchange (ISI) Foundation, Torino, Italy

[8]Laboratoire de Physique de l'Ecole Normale Supérieure de Lyon, CNRS UMR 5672, Lyon, France

[§]Corresponding author

Supplementary figure 4 – Boxplots showing the distributions of the number of final cases when the final attack rate is larger than 10%, according to the different scenarios and network types. The bottom and top of the rectangular boxes correspond to the 25th and 75th quantile of the distribution, the horizontal line to the median, and the ends of the whiskers give the 5th and 95th. Very short latency, very short infectiousness scenario: σ^{-1}=1 days, ν^{-1}=2 days and β=3.10^{-4} s^{-1}. Short latency, short infectiousness scenario: σ^{-1}=2 days, ν^{-1}=4 days and β=15.10^{-5} s^{-1}.

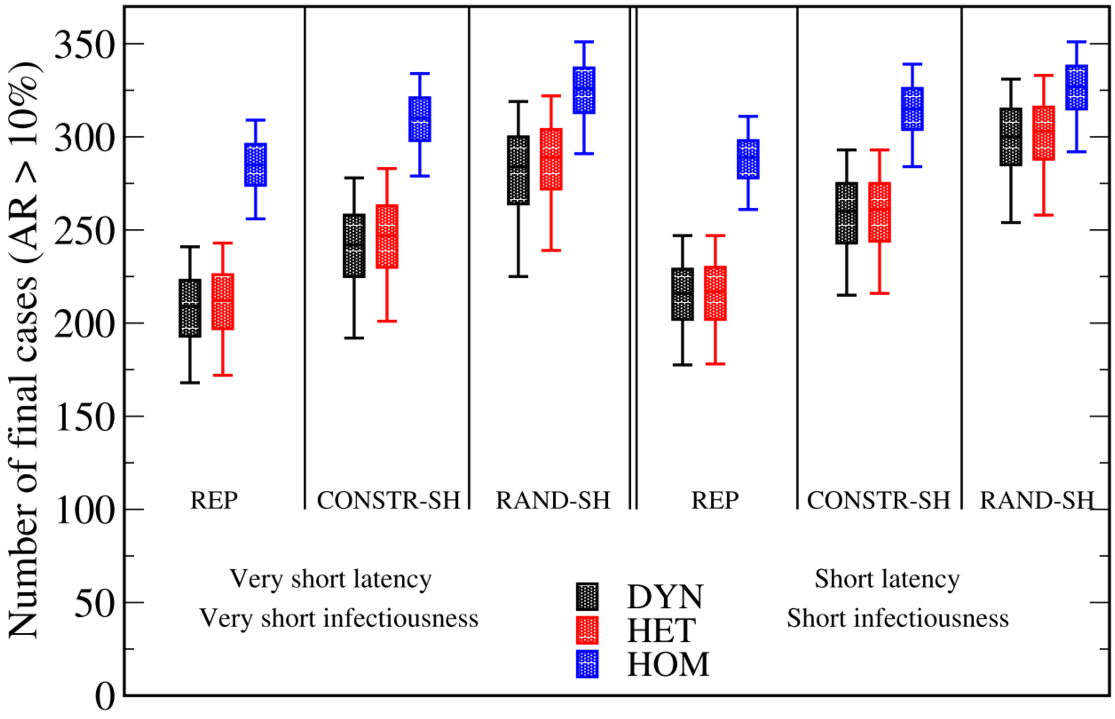

Supplement: Additional file 4 — Supplementary figure 4. Box plots showing the distributions of the number of final cases when the final attack rate is larger than 10%, according to the different scenarios and network types. [file 1741-7015-9-87-S4.PDF]
