# Supplementary material for: Simulation of an SEIR infectious disease model on the dynamic contact network of conference attendees
Source: BMC Med. 2011 Jul 19;9:87. doi: 10.1186/1741-7015-9-87 (PMC3162551; doi:10.1186/1741-7015-9-87)
Supplement: Additional file 5 — Supplementary table 2. Mean values, variances and 90% CI of the prevalence peak time tpeak according to the different scenarios and network types. [file 1741-7015-9-87-S5.PDF]

Simulation of a SEIR infectious disease model on the dynamic contact network of conference attendees

Additional file 5 – Supplementary table 2

Juliette Stehlé[1], Nicolas Voirin[2,3§], Alain Barrat[1,4], Ciro Cattuto[4], Vittoria Colizza[5,6,7], Lorenzo Isella[4], Corinne Régis[3], Jean-François Pinton[8], Nagham Khanafer[2,3], Wouter Van den Broeck[4] and Philippe Vanhems[2,3]

[1]Centre de Physique Théorique de Marseille, CNRS UMR 6207, Marseille, France
[2]Hospices Civils de Lyon, Hôpital Edouard Herriot, Service d'Hygiène, Epidémiologie et Prévention, Lyon, France
[3]Université de Lyon; université Lyon 1; CNRS UMR 5558, laboratoire de Biométrie et de Biologie Evolutive, Equipe Epidémiologie et Santé Publique, Lyon, France
[4]Data Science Laboratory, Institute for Scientific Interchange (ISI) Foundation, Torino, Italy
[5] INSERM, U707, Paris F-75012, France
[6] UPMC Université Paris 06, Faculté de Médecine Pierre et Marie Curie, UMR S 707, Paris F75012, France
[7]Computational Epidemiology Laboratory, Institute for Scientific Interchange (ISI) Foundation, Torino, Italy
[8]Laboratoire de Physique de l'Ecole Normale Supérieure de Lyon, CNRS UMR 5672, Lyon, France
[§]Corresponding author

Supplementary table 2 – Average values, variances and 90% confidence interval (90% CI) of the prevalence peak time t_{peak} according to the different scenarios and network types. Only runs with AR>10% are taken into account.

**Scenarios**	**Parameters**	**Network**	**Number of runs**	**$<t_{peak}>$ days**	**Variance**	**90% CI**
Very short latency<br>Very short infectiousness<br>REP	$1/\sigma = 1$ days<br>$1/\nu = 2$ days<br>$\beta = 3.10^{-4}\ s^{-1}$	DYN	2000	14.7	25	[8,23]
		HET	2000	14.3	24	[8,23]
		HOM	2000	14.3	14	[9,21]
Short latency<br>Short infectiousness<br>REP	$1/\sigma = 2$ days<br>$1/\nu = 4$ days<br>$\beta = 15.10^{-5}\ s^{-1}$	DYN	2000	28.1	84	[16,45]
		HET	2000	27.6	78	[16,43]
		HOM	2000	27.7	47	[18,41]
Very short latency<br>Very short infectiousness<br>RAND-SH	$1/\sigma = 1$ days<br>$1/\nu = 2$ days<br>$\beta = 3.10^{-4}\ s^{-1}$	DYN	2000	19.9	53	[11,33]
		HET	2000	19.2	46	[11,32]
		HOM	2000	17.8	25	[11,27]
Short latency<br>Short infectiousness<br>RAND-SH	$1/\sigma = 2$ days<br>$1/\nu = 4$ days<br>$\beta = 15.10^{-5}\ s^{-1}$	DYN	2000	39.6	168	[23,63]
		HET	2000	39.	148	[23,61]
		HOM	2000	36.7	108	[23,56]
Very short latency<br>Very short infectiousness<br>CONSTR-SH	$1/\sigma = 1$ days<br>$1/\nu = 2$ days<br>$\beta = 3.10^{-4}\ s^{-1}$	DYN	2000	15.9	31	[9,27]
		HET	2000	15.1	27	[9,25]
		HOM	2000	15.6	18	[10,24]
Short latency<br>Short infectiousness<br>CONSTR-SH	$1/\sigma = 2$ days<br>$1/\nu = 4$ days<br>$\beta = 15.10^{-5}\ s^{-1}$	DYN	2000	30.4	97	[17,47]
		HET	2000	30.4	101	[17,49]
		HOM	2000	31.5	71	[20,46]
